# HSF1 Protects Sepsis-Induced Acute Lung Injury by Inhibiting NLRP3 Inflammasome Activation

**DOI:** 10.3389/fimmu.2022.781003

**Published:** 2022-06-01

**Authors:** Xueyan Shi, Tao Li, Yanting Liu, Leijin Yin, Lan Xiao, Liyao Fu, Yaxi Zhu, Huan Chen, Kangkai Wang, Xianzhong Xiao, Huali Zhang, Sichuang Tan, Sipin Tan

**Affiliations:** ^1^ Sepsis Translational Medicine Key Laboratory of Hunan Province, Central South University, Changsha, China; ^2^ State Key Laboratory of Molecular Oncology, National Cancer Center/National Clinical Research, Center for Cancer/Cancer Hospital, Chinese Academy of Medical Sciences and Peking Union, Medical College, Beijing, China; ^3^ Department of Pathophysiology, Medical College of Jiaying University, Meizhou, China; ^4^ Department of Pathophysiology, Xiangya School of Medicine, Central South University, Changsha, China; ^5^ Department of Traditional Chinese Medicine, The Third Xiangya Hospital, Central South University, Changsha, China; ^6^ The Second Xiangya Hospital, Central South University, Changsha, China

**Keywords:** HSF1, sepsis, NLRP3 inflammasome, TRAF3, SGT1

## Abstract

As an important transcription factor, heat shock factor 1 (HSF1) plays an endogenous anti-inflammation role in the body and can alleviate multiple organ dysfunction caused by sepsis, which contributes to an uncontrolled inflammatory response. The NLRP3 inflammasome is a supramolecular complex that plays key roles in immune surveillance. Inflammation is accomplished by NLRP3 inflammasome activation, which leads to the proteolytic maturation of IL-1β and pyroptosis. However, whether HSF1 is involved in the activation of the NLRP3 inflammasome in septic acute lung injury (ALI) has not been reported. Here, we show that HSF1 suppresses NLRP3 inflammasome activation in transcriptional and post-translational modification levels. HSF1 can repress NLRP3 expression *via* inhibiting NF-κB phosphorylation. HSF1 can inhibit caspase-1 activation and IL-1β maturation *via* promoting NLRP3 ubiquitination. Our finding not only elucidates a novel mechanism for HSF1-mediated protection of septic ALI but also identifies new therapeutic targets for septic ALI and related diseases.

## Introduction

Sepsis refers to dysregulated host responses to infections that lead to life-threatening organ dysfunctions. Sepsis is increasing every year in China, and more than one million people die from sepsis annually, which occupies a large number of medical resources (1, 2). The uncontrolled inflammatory response and multiple organ dysfunction induced by sepsis are attributed to its high mortality rate. In sepsis, inflammatory cells are activated and release a large number of inflammatory mediators, such as tumor necrosis factor (TNF), interleukin-1, and oxygen free radicals, which cause serious damage to the tissues and body organs (3, 4). As one of the most vulnerable organs in sepsis, acute lung injury (ALI) can progress into acute respiratory distress syndrome, which finally leads to multiple organ dysfunction and death. Therefore, inhibition of the inflammatory response can protect sepsis-induced ALI and improve the survival of sepsis (5, 6).

Inflammasomes are cytoplasmic sensors that sense pathogen infection and tissue damage, leading to the maturation and release of pro-inflammatory cytokines such as IL-1β and IL-18. Currently, the best-characterized inflammasome is the NOD-like receptor (NLR)-family pyrin domain-containing 3 (NLRP3) inflammasome. The NLRP3 inflammasome is composed of a sensor NLRP3, an adaptor ASC (apoptosis-associated speck-like protein containing caspase activation and recruitment domain), and an effector pro-caspase-1 (7). NLRP3 inflammasome activation requires two signals. Triggered by pathogen-associated molecular patterns (PAMPs), TNF, and IL-1β, the first signal upregulates the mRNA expression of NLRP3 and pro-IL-1β by activating nuclear factor-κB (NF-κB). The second signal is activated by other stimuli such as pore-forming toxins, ATP, and particulate matter, which lead to inflammasome assembly and caspase-1 activation. Activated caspase-1 cuts pro-IL-1β and pro-IL-18 into their mature forms and secretes these inflammatory cytokines into extracellular space (8). Posttranslational modifications (PTMs) such as ubiquitination are crucial regulators of inflammasome assembly. Dysregulated inflammasomes are responsible for many chronic diseases such as Alzheimer’s disease and rheumatoid arthritis (9, 10). Further clarifying transcriptional and posttranslational regulation of inflammasomes such as ubiquitination will shed more light on various diseases (11, 12).

As a key transcription factor regulating heat shock or stress responses, heat shock factor 1 (HSF1) can bind to heat shock elements (HSEs) of various downstream target genes, showing different protective effects against cellular damage (13). Previous research has proved that HSF1 plays an important role in suppressing the inflammatory response. For example, HSF1 can reduce liver tissue damage and the development of alcohol-associated liver diseases by inhibiting NLRP3 inflammasome activation (14, 15). Also, HSF1 significantly alleviated lipopolysaccharide (LPS)-induced ALI by inhibiting oxidative stress and inflammatory response (16). However, whether HSF1 affects the NLRP3 inflammasome activation during sepsis-induced ALI and its molecular mechanisms remains unknown.

In our research, we demonstrated that septic lung tissue expressed NLRP3 inflammasome-related components and produced a large number of IL-1β and IL-18. The high expressions of IL-1β and IL-18 were confirmed in septic patients. HSF1 inhibited the activation of NLRP3 inflammasome and the secretion of IL-1β and IL-18 *in vitro* and *in vivo*. Our further experiments proved that HSF1 inhibited the activation of the NLRP3 inflammasome by inhibiting the NF-κB signaling pathway and promoting ubiquitination of NLRP3 at transcriptional and post-translational modification levels, respectively. SGT1 and TRAF3 decreased consistently in septic patients, which serves as extra proof that they can be candidate therapeutic target molecules in sepsis.

## Materials and Methods

### Animal

HSF1 knockout mice were donated by Benjamin’s laboratory; wild-type (HSF1^+/+^) mice of the same age, strain, and sex were used as control. The protocols for animal breeding and experiments were approved by the Institutional Animal Care and Use Committee of Central South University, approval Number 2018sydw077. All animal experiments were conducted in the Department of Zoology, Central South University.

### Clinical Sepsis Patient Specimens

Sepsis was diagnosed according to the definition of sepsis 3.0, and the Sequential Organ Failure Assessment (SOFA) score was ≥2 points. Each of the six SOFA parameters reflects the function of the organ systems (respiratory, cardiovascular, renal, neurological, hepatic, and hematological) with a score of 0 to 4. The patients had different degrees of lung injury; most of the patients had symptoms such as cough, sputum, and fever. The collection of specimens was approved by the ethical review of the Third Xiangya Hospital of Central South University, Approval Number 2019-S548. The subjects were informed of the collection of this specimen and provided signed informed consent.

### Antibodies

The following antibodies were used: rabbit anti-NLRP3 monoclonal antibody (Cell Signaling Technology, Danvers, MA, USA, dilution 1:1,000); mouse anti-IL-1β monoclonal antibody (Cell Signaling Technology, USA, dilution 1:1,000); mouse anti-Ub monoclonal antibody (Santa Cruz, Dallas, TX, USA, dilution 1:300); rabbit anti-SUGT1 polyclonal antibody (ProteinTech, Chicago, IL, USA, dilution 1:1,000); rabbit anti-HSF1 polyclonal antibody (Cell Signaling Technology, USA, dilution 1:1,000); rabbit anti-pro Caspase-1+p10+p20 monoclonal antibody (abcam, Cambridge, UK, dilution 1:1,000); rabbit anti-IgG (Cell Signaling Technology, USA, dilution 1 μg); mouse antiglyceraldehyde-3-phosphate dehydrogenase (GAPDH) monoclonal antibody (Cell Signaling Technology, USA, dilution 1:5,000); and mouse anti-ACTIN monoclonal antibody (Cell Signaling Technology, USA, dilution 1:1,000).

### Cecal Ligation and Puncture Model

Mice were anesthetized with isoflurane inhalation; the skin and muscle layers along the midline of the lower abdomen were cut; the abdominal cavity was exposed to find the cecum; the end of the cecum 1/3 was ligated with line 3; the distal end of the cecum was punctured (through the cecum) with a size 7 needle, squeezing out a little stool gently; then the cecum is gently returned to the abdominal cavity; the peritoneum and the muscular layer are sterilized; and the muscular layer and skin are sewn up and sterilized again. Mice were rehydrated by subcutaneous injection of preheated saline into the back. They were kept warm after the operation, and the general condition of the mice was observed. After the mice were modeled, the state of the mice was observed, including 7 indicators of appearance, consciousness level, activity, stimulus response, eye condition, respiratory rate, and respiratory quality. Each indicator was graded from 0 to 4 points, and the scores of the 7 indicators were added together. The highest score was 28 points, and the dead mice were recorded as having the highest score. H&E staining was used to detect the lung histopathology of mice, and the mice in the cecal ligation and puncture (CLP) group had secondary lung injury, which manifested as a large number of inflammatory cells, debris, and red blood cell (RBC) infiltration in the alveolar cavity and interalveolar septum, destruction of alveolar structure, and obvious edema and thickening of the alveolar septum. The results showed that the CLP-induced sepsis model was successfully replicated and induced lung injury.

### Real-Time Quantitative PCR

Total RNA was purified from cells using TRIzol^®^ Reagent (Gibco, Grand Island, NY, USA) according to the manufacturer’s instructions. Complementary DNA (cDNA) was synthesized using total RNA as a template with PrimeScript^®^ RTreagent Kit and gDNA Eraser kit (TaKaRa Bio, Siga, Japan). Diluted cDNAs were subjected to qPCR analysis using SYBR^®^ Green Quantitative PCR kit (TaKaRa Bio, Siga, Japan). Relative mRNA levels were calculated after normalization to actin using the ΔCt method. The PCR primer sequences used in this paper are listed in **Table 1**. Primer is designed by Primer5.0 online software, and biosynthesis is performed by TSINGKE (Changsha, China).

**Table 1:** PCR primer sequence.

Gene	Forward sequence	Reverse sequence
Caspase-1	TGCCGTGGAGAGAAACAAGG	CCCCTGACAGGATGTCTCCA
NLRP3	GACACGAGTCCTGGTGACTT	GGCTTAGGTCCACACAGAAAG
SGT1	AGAGAGAGTTGTCTGCTTTGGT	CTTTTCCCATCTCACCGCCT
β-Actin	CATTGCTGACAGGATGCAGAAGG	TGCTGGAAGGTGGACAGTGAGG
IL-18	TAAGAGGACTGGCTGTGACC	TGGCAAGCA AGA AAGTGTCC
IL-1β	TCTTTGAAGTTGACGGACCC	TGAGTGATACTGCCTGCCTG
GAPDH	CATCACTGCCACCCAGAAGACTG	ATGCCAGTGAGCTTCCCGTTCAG
HSF1-KO	TCTCCTGTCCTGTGTGCCTAGC	CAGGTCAACTGCCTACACAGACC
NEO	AGGACATAGCGTTGGCTACCCGTG	GCCTGCTATTGTCTTCCCAATCC

### Western Blotting

Tissue and cell proteins were lysed with radioimmunoprecipitation assay (RIPA) lysates (P0013, Beyotime, Shanghai, China), the supernatant was collected after ultrasound and centrifugation, protein concentrations were measured using a bicinchoninic acid (BCA) protein quantification kit (98L001100, Beijing Dingguo Changsheng Biotechnology Co., Ltd., Beijing, China), and the same amount of protein was mixed with 5× sodium dodecyl sulfate (SDS) loading buffer and heated for 10 min at 95°C. The pyrolysis products were separated by 10% SDS–polyacrylamide gel electrophoresis (SDS-PAGE), transferred to polyvinylidene difluoride (PVDF) membrane, sealed in TBST containing 5% skim milk powder for 2 h, and incubated overnight with the corresponding primary antibody. After being washed with TBST three times, horseradish peroxidase-labeled anti-rabbit or anti-mouse IgG was used as the secondary antibody to seal for 1.5 h, and the immune response bands were observed by the chemiluminescence imaging system (Aplegen, Pleasanton, CA, USA). Anti-GAPDH or anti-actin antibody was used to normalize equal amounts of proteins.

### Enzyme-Linked Immunosorbent Assay

The serum levels of IL-1β (CSB-E08054m, CUSABIO, Wuhan, China) and IL-18 (CSB-E04609m, CUSABIO, China) in each group were measured using a mouse ELISA kit. A human ELISA kit was used to detect serum levels of IL-1β (CSB-E08053h, CUSABIO, China), IL-18 (CSB-E07450h, CUSABIO, China), and SGT1 (69-32967, MSKBIO, Wuhan, China) in sepsis patients and normal people. All the experimental procedures were carried out according to the kit instructions.

### Isolation of Primary Mouse Peritoneal Macrophages

Mice were intraperitoneally injected with 3 ml of 3% thioglycolate broth medium and sacrificed *via* cervical dislocation after 4 days. After being immersed in 75% alcohol for 10 min, the sacrificed mice were placed in a supine position on an ultra-clean workbench where their abdominal skins were cut open and then flushed twice with 5 ml of pre-chilled Roswell Park Memorial Institute (RPMI) 1640 medium (supplemented with 10 U/ml of penicillin and 100 μg/ml of streptomycin), followed by a collection of their peritoneal lavage fluid (PLF). After centrifugation (1,000 rpm, 5 min), the resulting supernatant of the PLF was discarded, and the pellet was resuspended with 5 ml of 1× RBC Lysis Buffer (555899, BD Biosciences, San Jose, CA, USA) and incubated in an ice bath for 10 min. After a second centrifugation step, the resulting supernatant was discarded again, and the cell pellet was resuspended and plated in RPMI 1640 medium containing 10% fetal bovine serum (FBS) for cultivation.

### Electrophoretic Mobility Shift Assay

RAW264.7 cells were treated at 43°C in a 10-cm cell culture dish for 60 min and immediately by the extraction of nuclear proteins (P0027, Beyotime, China). After the protein concentration was determined *via* the BCA assay, the nuclear protein samples were stored at −80°C in a freezer until use. DNA probes were generated according to the HSE sites as double-stranded (**Tables 2** and **3**). After being validated with nucleotide blast, the probe sequences were sent to Sangon Biotech (Shanghai, China) Co., Ltd., for probe synthesis. Electrophoretic mobility shift assay (EMSA) was performed in accordance with instructions provided in the kit (20148, Thermo Scientific™, Waltham, MA, USA), and the results were visualized using a chemiluminescence imaging system (Aplegen, USA).

**Table 2:** SGT1 probe sequence.

Probe	Probe sequence
SGT1 Labeled probe F	5′-AGAATTCGCAGGAAACTTCCTAGTGTGAT-3′
SGT1 Labeled probe R	5′-ATCACACTAGGAAGTTTCCTGCGAATTCT-3′
SGT1 Competitive probe F	5′-AGAATTCGCAGGAAACTTCCTAGTGTGAT-3′
SGT1 Competitive probe R	5′-ATCACACTAGGAAGTTTCCTGCGAATTCT-3′
SGT1 Mutant probe F	5′-AGCAGTCACAGTCAACGTCATATTGAGAT-3′
SGT1 Mutant probe R	5′-ATCTCAATATGACGTTGACTGTGACTGCT-3′

**Table 3:** TRAF3 probe sequence.

Probe	Probe sequence
TRAF3 Labeled probe F	5′-ATTCCTGAGAGAAATTTCTTTGTCCAGA-3′
TRAF3 Labeled probe R	5′-TCTGGACAAAGAAATTTCTCTCAGGAAT-3′
TRAF3 Competitive probe F	5′-CGTCATGCGCTAGATCGCTGTGTATATA-3′
TRAF3 Competitive probe R	5′-TATATACACAGCGATCTAGCGCATGACG-3′
TRAF3 Mutant probe F	5′-ATTCCTGAGAGAAATTTCTTTGTCCAGA-3′
TRAF3 Mutant probe R	5′-TCTGGACAAAGAAATTTCTCTCAGGAAT-3′

### Dual-Luciferase Reporter Assay

Luciferase reporter plasmids containing SGT1 and TRAF3 were constructed (Sangon Biotech, Shanghai, China). The plasmids were extracted (D6950-01, Omega, Doraville, GA, USA), their concentration was determined, and the DNA was stored at −20°C for subsequent experiments. HEK293 cells were inoculated in a 24-well plate and grown to about 70% to 90% confluence before being co-transfected (L3000015, Invitrogen™, Carlsbad, CA, USA) with pcDNA3.1, HSF1-constitutive plasmids, luciferase reporter plasmids, PGL-basic, and pRL-TK. After 48 h of transfection, the cell lysate was extracted and tested using a dual-luciferase reporter assay kit (E1910, Promega, Madison, WI, USA) and loaded onto a luminometer for the measurement of luminescence (Synergy^HI^ Microplate Reader, BioTek, Winooski, VT, USA).

### Co-Immunoprecipitation

Protein A/G Mix Magnetic Beads (50 µl) (LSKMAGAG10, Millipore, Billerica, MA, USA) were pipetted into a 2-ml microcentrifuge tube and washed three times with PBST (phosphate-buffered saline (PBS) containing 0.01% Tween^®^20 detergent). After being resuspended with PBS, primary antibodies NLRP3, SGT1, and IgG (rabbit IgG) measuring 1.0–10.0 µl were added and then incubated at room temperature (23°C–26°C) for 1 h. After being washed three times with PBST for 10 min each time, 150 µg of protein sample was added to the antibody-coated magnetic beads, which were then incubated overnight with agitation at 4°C. Magnetic beads were washed three times with PBST for 10 min each, and 1× SDS elution buffer was added, followed by heating at 85°C for 10 min. Subsequently, the supernatant was harvested *via* aspiration, and 5× SDS loading buffer was added, followed by heating at 95°C for 10 min, before performing SDS-PAGE.

### Ubiquitination Assay

Total cellular protein measuring 150 μg was added to a 1.5-ml microcentrifuge tube, and primary antibodies NLRP3 (#15101, Cell Signaling Technology, USA) and IgG (#2729, Cell Signaling Technology, USA) measuring were added and incubated for 1 h at 4°C. The resuspended volume of Protein A/G plus-agarose (sc-2003, Santa Cruz, USA) measuring 20 μl was added and incubated at 4°C on a rotating device for 1 h overnight. Immunoprecipitates were collected by centrifugation at 2,500 rpm for 5 min at 4°C, and the pellet was washed four times with PBS, each time repeating the above centrifugation steps. After the final wash, the supernatant was aspirated and discarded, and the pellet was resuspended in a 1× electrophoresis sample buffer. Samples were boiled for 2–3 min and analyzed by SDS-PAGE. Proteins were transferred onto methanol-activated 0.2-μm PVDF membranes. Membranes were washed three times in TBST and blocked in 5% non-fat dried milk in TBST. Membranes were then incubated overnight with an anti-Ub antibody.

### Statistical Analysis

Results are expressed as means ± SEM. All data were analyzed using PASW Statistics 21.0. Student’s t-test was applied for comparison between two groups and one-way ANOVA for multiple comparisons. Survival data were analyzed using the log-rank test. Differences were considered statistically significant when p < 0.05.

## Results

### HSF1 Alleviated Sepsis-Induced Acute Lung Injury in Mice

**Figure 1:**
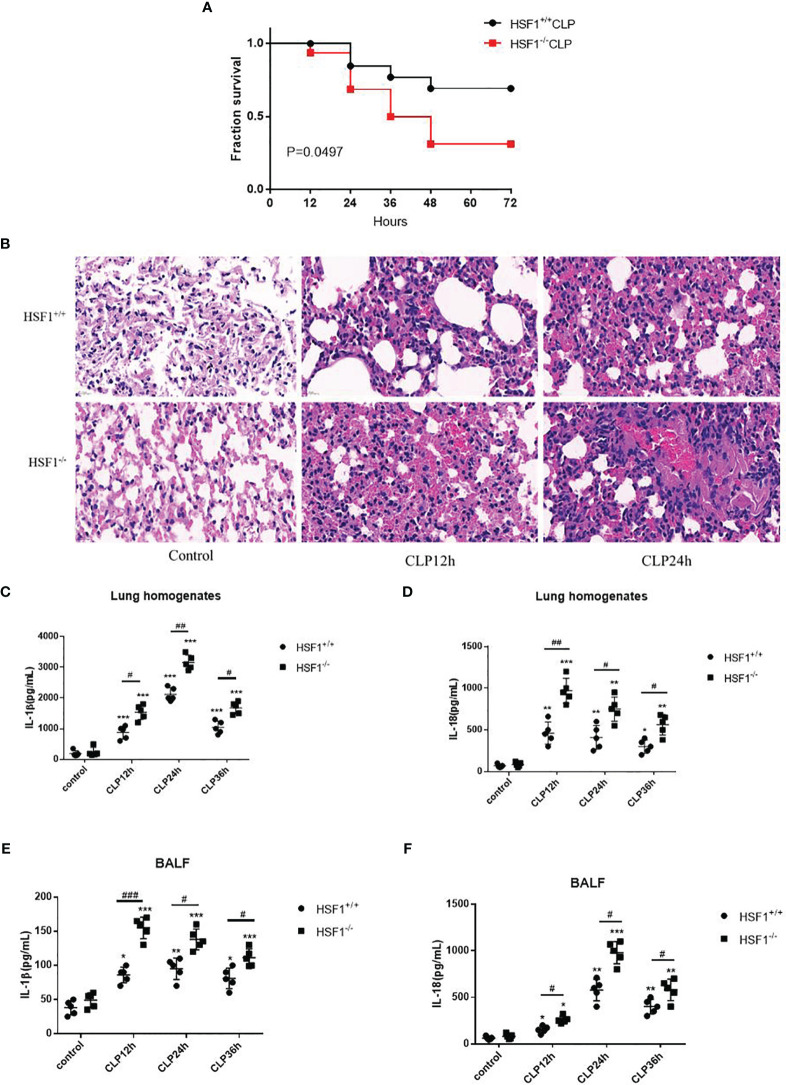
HSF1 ameliorates pulmonary inflammation in sepsis-induced ALI. **(A)** The survival rate of HSF1^−/−^ and HSF1^+/+^ mice after CLP. **(B)** After 12- and 24-h CLP interventions, representative pictures of lung tissue sections were stained with H&E. **(C–F)** The expression of IL-1β and lL-18 in lung homogenate and lung BALF after CLP operation was monitored by ELISA. Data are representative or means (SD) of at least three independent experiments. Statistical analyses were determined using a Student’s *t*-test and one-way ANOVA. ^*^p < 0.05, ^**^p < 0.01, ^***^p < 0.001 versus control group; ^#^p < 0.05, ^##^p < 0.01, ^###^p < 0.001 versus HSF1^+/+^ CLP group. ALI, acute lung injury; CLP, cecal ligation and puncture; BALF, bronchoalveolar lavage fluid.

In order to demonstrate the protective effects of HSF1 against sepsis-induced lung injury, CLP was used to prepare the sepsis model, and the survival curve was plotted. As shown in **Figure 1A**, the survival rate of HSF1 knockout (HSF1^−/−^) mice was significantly lower than that of HSF1^+/+^ mice. Subsequently, the histological examination of lung tissues after CLP was detected. H&E staining showed hyperemia and edema in the alveolar cavity, the alveolar septa were significantly widened, accompanied by a large number of inflammatory cells infiltration, and the HSF1^−/−^ group was significantly more serious than the HSF1^+/+^ group (**Figure 1B**). In addition, ELISA showed that the expression of inflammatory cytokines IL-1β and IL-18 in lung tissue homogenate and alveolar lavage fluid of mice after CLP was significantly increased, and the HSF1^−/−^ group was significantly higher than the HSF1^+/+^ group (**Figures 1C–F**). The above results indicated that HSF1 can significantly alleviate CLP-induced ALI and improve the survival rate of septic mice by inhibiting the inflammatory response.

### HSF1 Inhibited NLRP3 Inflammasome Activation

**Figure 2:**
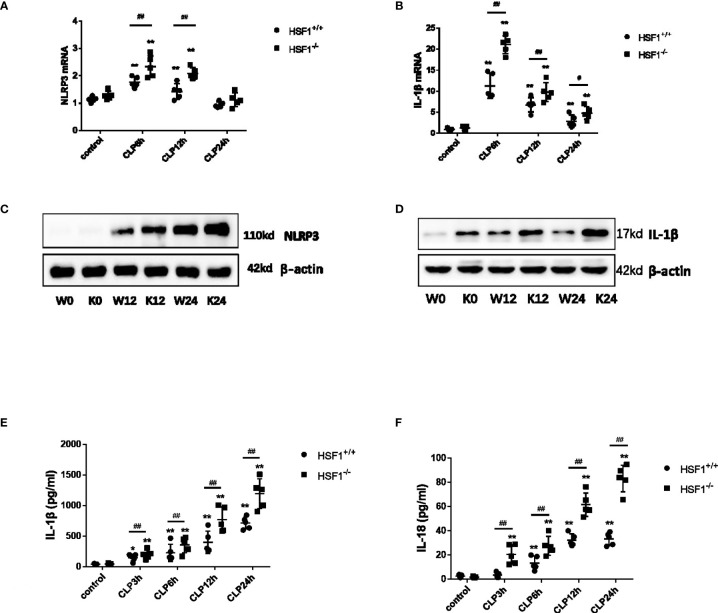
HSF1 suppresses the activation of NLRP3 inflammasome in lung tissue. **(A, B)** HSF1^−/−^ and HSF1^+/+^ mice after CLP surgery; the gene expressions of *NLRP3* and *IL-1β* in the lung tissue were assessed by qPCR. **(C, D)** The protein levels of NLRP3 and IL-1β were measured by Western blotting. **(E, F)** Serum levels of IL-1β and IL-18 were detected by ELISA. Data are representative or means (SD) of three independent experiments. Statistical analyses were performed using one-way ANOVA. ^*^p < 0.05, ^**^p < 0.01 versus control group; ^#^p < 0.05, ^##^p < 0.01 versus HSF1^+/+^ CLP group. CLP, cecal ligation and puncture.

To clarify the NLRP3 inflammasome activation in sepsis, we performed CLP on mice to observe the NLRP3 inflammasome expression in lung tissue. It is found that the mRNA and protein levels of NLRP3 and IL-1β were both increased in the HSF1^+/+^ and HSF1^−/−^ groups after CLP, but the enhanced expression was significantly higher in the HSF1^−/−^ group (**Figures 2A–D**). The inflammasome-related molecule expression was also detected in lung tissue. CLP intervention significantly increased the production of IL-1β and IL-18 in serum in both the HSF1^−/−^ and HSF1^+/+^ groups, with a significantly higher cytokine increase in the HSF1^−/−^ group (**Figures 2E, F**).

**Figure 3:**
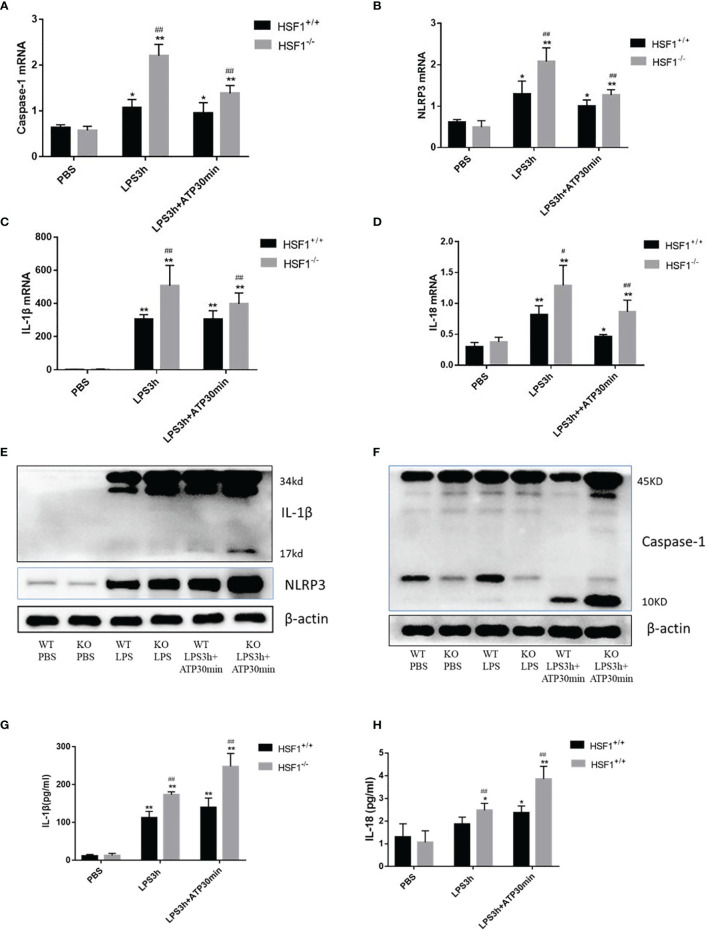
HSF1 represses the activation of NLRP3 inflammasome in peritoneal primary macrophages. **(A–D)** Peritoneal primary macrophages were isolated from HSF1^−/−^ mice and HSF1^+/+^ mice. A total of 1 day later, the cells were stimulated with LPS (100 ng/ml) for 3 h, followed by ATP (2.5 mM) for 30 min. The mRNA levels of *NLRP3*, *caspase-1*, *IL-1β*, and IL-18 in peritoneal primary macrophages cells were analyzed *via* qPCR. **(E, F)** The protein levels of *NLRP3*, *caspase-1*, and *IL-1β* were measured by Western blotting. **(G, H)** Cell supernatant levels of IL-1β and IL-18 were detected by ELISA. Data are representative or means (SD) of at least three independent experiments. Statistical analyses were determined using one-way ANOVA. ^*^p < 0.05, ^**^p < 0.01 versus control group; ^#^p < 0.05, ^##^p < 0.01 versus HSF1^+/+^ CLP group. LPS, lipopolysaccharide; CLP, cecal ligation and puncture.

Peritoneal primary macrophages were isolated from HSF1^−/−^ and HSF1^+/+^ mice. The peritoneal primary macrophages were stimulated with ATP, an activator of the NLRP3 inflammasome (17). A total of 1 day later, the cells were stimulated with LPS for 3 h, followed by ATP (2.5 mM) for 30 min. As shown in **Figure 3**, the mRNA expressions of NLRP3, caspase-1, IL-18, and IL-1β in macrophages were sharply increased in the HSF1^−/−^ group than in the HSF1^+/+^ group after LPS and ATP stimulations. The protein expressions of NLRP3, activated caspase-1, and mature IL-1β also increased much higher in the HSF1^−/−^ group. The secretion of mature IL-1β and IL-18 in supernatants also increased much higher in HSF1^−/−^ mice. These results collectively indicate that HSF1 can inhibit the activation of NLRP3 inflammasome in the lung tissue of septic mice and primary peritoneal macrophages.

### HSF1 Inhibited Lipopolysaccharide-Induced NLRP3 Activation *via* Suppressing NF-κB Signaling Pathway

**Figure 4:**
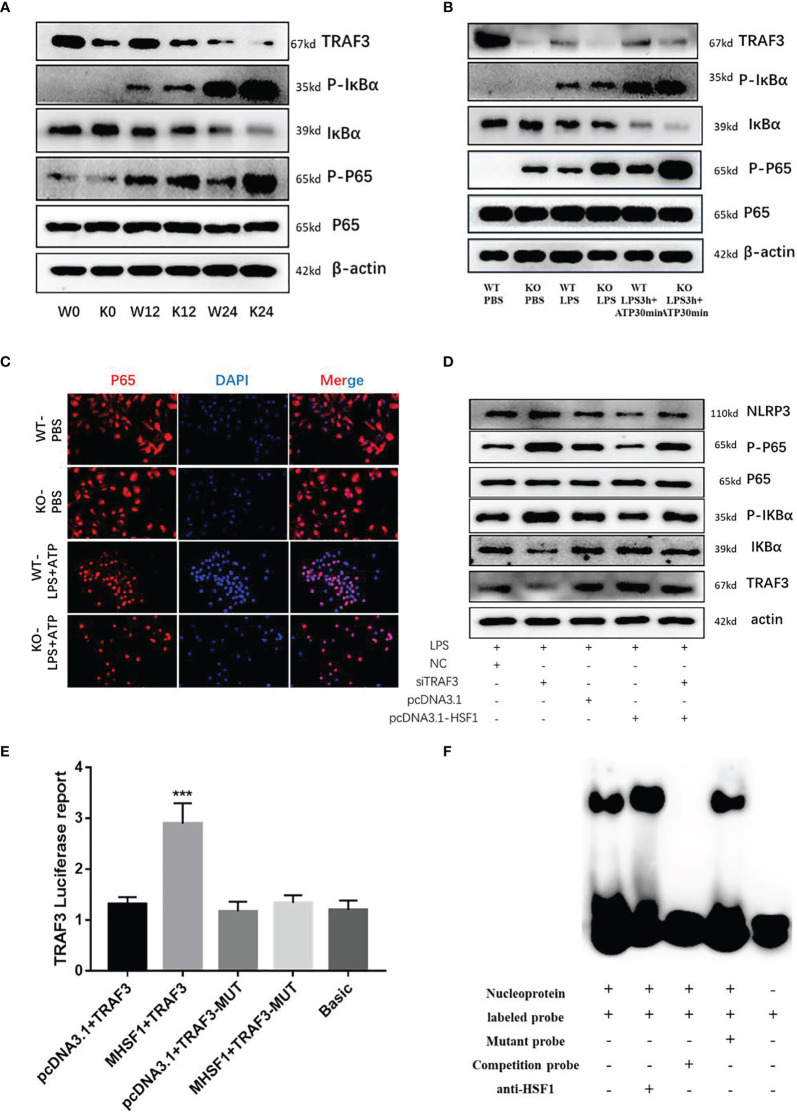
HSF1 inhibits the activation of NF-κB signaling pathway by upregulating TRAF3 expression. **(A)** Western blotting detected the expression of NF-κB pathway-related proteins in the lung tissues of septic mice. **(B)** Western blotting detected the expression of NF-κB pathway-related proteins in LPS3h (100 ng/ml), and ATP (2.5 mM) induced primary peritoneal macrophages of mice. **(C)** Immunofluorescence was used to detect the nucleation of P65 under different treatment conditions. **(D)** Western blotting detected the expression of NF-κB pathway-related proteins in RAW264.7 cells transfected with pcDNA3.1-HSF1 and siTRAF3. **(E)** The transcriptional regulation of HSF1 on TRAF3 was detected by dual-luciferase reporter genes. **(F)** The combination of HSF1 and TRAF3 was detected by EMSA. Data are representative or means (SD) of at least three independent experiments. Statistical analyses were determined using a Student’s *t*-test. ^***^p < 0.001, compared with the control (pcDNA3.1+TRAF3). LPS, lipopolysaccharide; EMSA, electrophoretic mobility shift assay.

NF-κB activation is required for NLRP3 and pro-IL-1β production (18); therefore, we investigated whether HSF1 impaired LPS-induced NF-κB activation. TRAF3 is an important class of intracellular signal transduction factors involved in the signal transduction of a variety of receptor families, including the TNF receptor (TNFR) family, Toll-like receptor (TLR) family, interleukin-1 receptor (IL-1R) family, and RIG-I-like receptor (RLR) family, and plays an important role in innate and acquired immunity. When the receptor is activated, TRAF3 directly or indirectly recruits the intracellular domain of the downstream receptor, participates in signal transduction, and ultimately inhibits the NF-κB signaling pathway, which is involved in the occurrence and development of inflammatory immune diseases (19, 20). To further investigate the role of HSF1 in the NF-κB signaling pathway in septic ALI, we detected the expression of NF-κB related proteins TRAF3, p-IκBα, and p-p65 in the lung tissues of septic mice. As shown in **Figure 4**, compared with the control group, TRAF3 and IκBα in the CLP group were decreased, while the expressions of p-IκBα and p-p65 were increased. Compared with the HSF1^+/+^ group, the expressions of TRAF3 and IκBα in the HSF1^−/−^ group were decreased more obviously, and the expressions of p-IκBα and p-p65 were increased more obviously.

Similarly, peritoneal primary macrophages were isolated from HSF1^−/−^ and HSF1^+/+^ mice. The cells were stimulated with LPS and ATP as described previously. We detected the expression of TRAF3, p-IκBα, and p-p65, and the results were consistent with those in the lung tissue of septic mice. Immunofluorescence detected the localization of phosphorylated P65 in primary peritoneal macrophages, compared with the control group, the fluorescence intensity of P65 in the LPS+ATP treatment group was increased while increased in the HSF1^−/−^ group compared with the HSF1^+/+^ group. The results suggested that the activation of the NF-κB signaling pathway was increased in the HSF1^−/−^ group. These results indicated that HSF1 can effectively inhibit the activation of the NF-κB signaling pathway in the lung tissues of septic mice and primary peritoneal macrophages induced by LPS.

**Figure 5:**
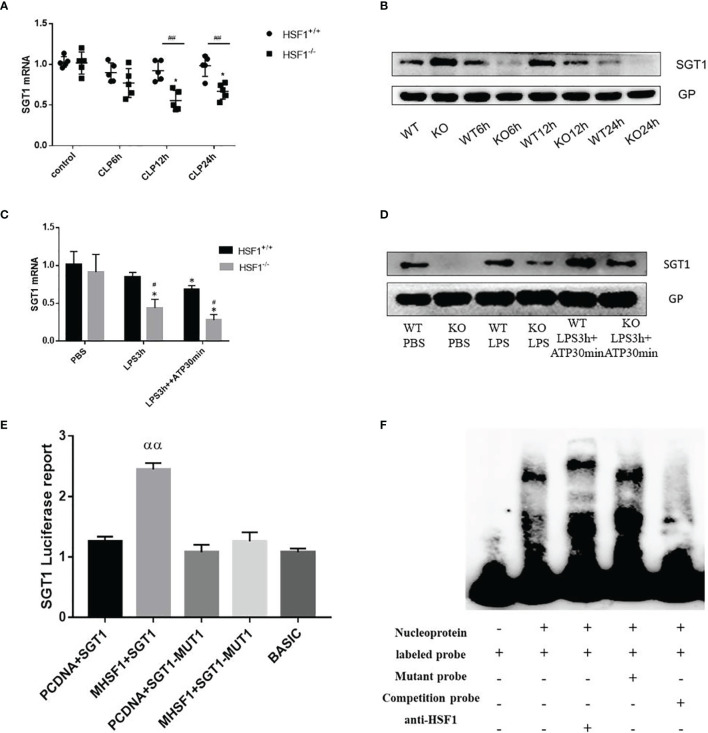
The influence of HSF1 on the transcriptional regulation of SGT1. **(A, B)** The expression of SGT1 in lung tissues of septic mice was detected by PCR and Western blotting. **(C, D)** The expression of SGT1 in primary peritoneal macrophages was detected by PCR and Western blotting. **(E)** The transcriptional regulation of HSF1 on SGT1 was detected by dual-luciferase reporter genes. **(F)** The combination of HSF1 and SGT1 was detected by EMSA. Data are representative or means (SD) of at least three independent experiments. Statistical analyses were determined using a Student’s *t*-test and one-way ANOVA. ^*^p < 0.05, versus control group; ^#^p < 0.05, ^##^p < 0.01, versus HSF1^+/+^ CLP; ^αα^P<0.01, versus PCDNA+SGT1. EMSA, electrophoretic mobility shift assay; CLP, cecal ligation and puncture.

TRAF3 plays an important role in the activation of the NF-κB signaling pathway, and TRAF3 expression is decreased in both the lung tissues of septic mice and the primary peritoneal macrophages of LPS-stimulated. The decrease was more obvious in the HSF1^−/−^ group, suggesting a certain correlation between TRAF3 and HSF1. In order to investigate whether HSF1 has a transcriptional regulation effect on TRAF3, we used the Jaspar database (http://jaspar.genereg.net/) to predict the presence of HSF1 transcriptional binding elements in the TRAF3 promoter region (21). As shown in **Figure 5**, the dual-luciferase reporter genes showed that the transcriptional activity of TRAF3 was significantly increased in the cells transfected with HSF1 composed of activated plasmid, while the transcriptional activity of the mutant TRAF3 was significantly decreased. Furthermore, the EMSA showed that the activation of HSF1 significantly increased the binding between HSF1 and TRAF3 DNA promoter region. At the same time, we co-transfected pcDNA3.1-HSF1 and siTRAF3 to detect the expression of NF-κB signaling pathway-related proteins and found that TRAF3 interference can reverse the inhibition of HSF1 on the NF-κB signaling pathway. These results suggest that HSF1 inhibited the NF-κB signaling pathway by upregulating TRAF3 expression, thereby inhibiting the production of NLRP3 at the transcriptional level and ultimately inhibiting the activation of the NLRP3 inflammasome.

### HSF1 Inhibited the NLRP3 Inflammasome Activation by Increasing NLRP3 Ubiquitination

Diverse PTMs determine the active state of NLRP3, among which polyubiquitination is critical for subsequent NLRP3 activation. FBXL2 is an SCF (SKP1-cullin-F-box) E3 ligase complex subunit that can recognize Trp73 in NLRP3 and target it for ubiquitination and proteasomal degradation (22). This finding established that the E3 ligase is required for NLRP3 ubiquitination-dependent activation, indicating that the SCF complex is most likely a key E3 ligase responsible for NLRP3 ubiquitination-dependent degradation in sepsis development. SGT1 (a suppressor of the G2 allele of SKP1) is a ubiquitin ligase-associated protein that associates with SKP1 and CUL1, subunits of the SCF ubiquitin ligase complex (23). Jaspar database (http://jaspar.genereg.net/) prediction identified the presence of HSF1 transcriptional binding elements in the SGT1 promoter region. Then we hypothesized that SGT1 may be involved in HSF1-regulated ubiquitination of NLRP3. We found that compared with the control group, the mRNA, and protein expression of SGT1 after CLP were decreased. Compared with the HSF1^+/+^ group, the expression of SGT1 in the HSF1^−/−^ group decreased more obviously. Similarly, we extracted primary peritoneal macrophages to detect the expression of SGT1, and the results were consistent with those in the lung tissue of septic mice. These results indicated that SGT1 expression was decreased in both septic mice and primary peritoneal macrophages, and the knockout of HSF1 could inhibit the expression of SGT1 (**Figure 5**).

The above findings suggest that HSF1 may be involved in the activation of the NLRP3 inflammasome by affecting the expression of SGT1. To prove whether HSF1 has a transcriptional regulation effect on SGT1, as shown in **Figure 5**, the dual-luciferase reporter genes showed that the transcriptional activity of SGT1 was significantly increased in the cells transfected with HSF1 composed of activated plasmid, while the transcriptional activity of the mutant SGT1 was significantly decreased. EMSA showed that HSF1 activation significantly increased the binding of HSF1 to the SGT1 promoter region. These results suggest that HSF1 can bind the HSE in the SGT1 promoter region to promote the transcription expression of SGT1.

**Figure 6:**
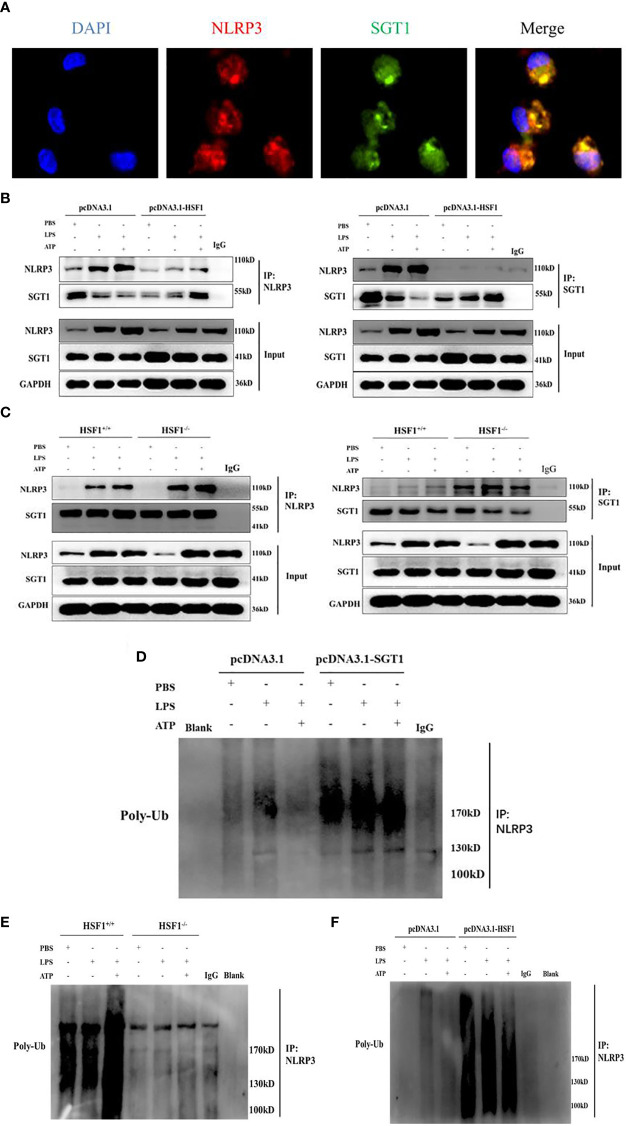
HSF1 inhibited the NLRP3 inflammasome activation by increasing the ubiquitination of NLRP3. **(A)** Expression and localization of NLRP3 and SGT1 in primary peritoneal macrophages were detected by immunofluorescence. Green and red colors represent SGT1 and NLRP3, respectively; blue represents nuclei. *Merge* (yellow) in the overlay images indicates co-localization. **(B)** The effects of HSF1 overexpression on the interaction between NLRP3 and SGT1 in LPS3h (1 μg/ml) and ATP (5.0 mM) induced RAW264.7 cells. **(C)** The effects of HSF1 knockout on the interaction between NLRP3 and SGT1 in LPS3h (100 ng/ml) and ATP (2.5 mM) induced primary peritoneal macrophages. **(D)** The effect of SGT1 overexpression on the ubiquitination of NLRP3 in LPS3h (1 μg/ml) and ATP (5.0 mM) induced RAW264.7 cells. **(E)** The effect of HSF1 overexpression on the ubiquitination of NLRP3 in LPS3h (1 μg/ml) and ATP (5.0 mM) induced RAW264.7 cells. **(F)** The effect of HSF1 knockout on the ubiquitination of NLRP3 in LPS3h (100 ng/ml) and ATP (2.5 mM) induced primary peritoneal macrophages. Data are representative or means (SD) of at least three independent experiments.

Protein interaction prediction from database String (https://string-db.org/) suggested that there may be interactions between SGT1 and NLRP3. To prove this hypothesis, we extracted primary peritoneal macrophages from mice and stimulated them with LPS+ATP for immunofluorescence co-localization. As shown in **Figure 6**, SGT1 and NLRP3 were found to co-locate in the cytoplasm. Immunoprecipitation showed that SGT1 protein in the cytoplasm could be precipitated by an anti-NLRP3 antibody. Similarly, anti-SGT1 antibodies precipitate the NLRP3 protein in the cytoplasm successfully. The expression of SGT1 was increased and the expression of NLRP3 was decreased after HSF1 overexpression. In contrast, the expression of SGT1 was reduced and the expression of NLRP3 was increased after HSF1 knockout.

SGT1 can interact with a variety of proteins such as certain SCF ubiquitin ligases (23), so we hypothesize that SGT1 may be involved in the ubiquitination of NLRP3 inflammasome. As shown in **Figure 6**, the ubiquitination of NLRP3 was significantly increased after SGT1 and HSF1 overexpression. However, in the primary peritoneal macrophages of the HSF1^−/−^ group, the ubiquitination of NLRP3 was significantly reduced compared with the HSF1^+/+^ group. These results suggest that HSF1 may inhibit the activation of NLRP3 inflammasome through SGT1-mediated ubiquitination of NLRP3.

### Increased IL-1β and IL-18 and Decreased TRAF3 and SGT1 Are Detected in Septic Patients

**Figure 7:**
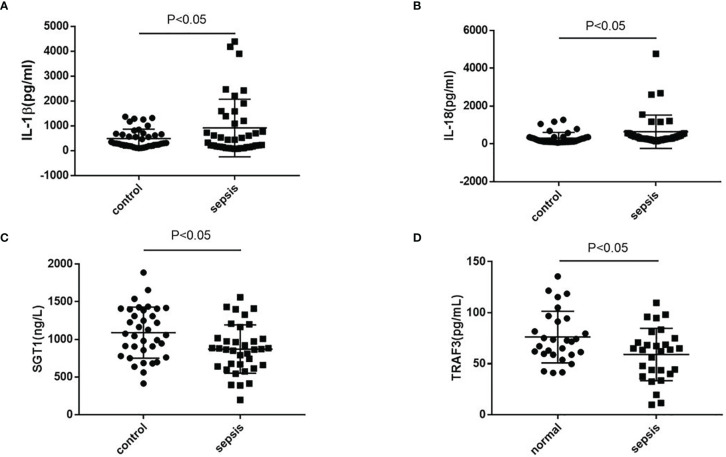
The expression of NLRP3 inflammasome-related genes in serum of patients with clinical sepsis. **(A–D)** The expression of IL-1β, IL-18, TRAF3, and SGT1 in serum of patients with clinical sepsis was detected by ELISA. Data are representative or means (SD) of at least three independent experiments. Statistical analyses were determined using a Student’s *t*-test.

The serum of septic patients was collected to further clarify NLRP3 inflammasome activation. The serum levels of IL-1β and IL-18 from septic patients and healthy controls (HCs) were detected. As shown in **Figure 7**, compared with HCs, septic patients had a higher expression of IL-1β and IL-18. Further detection of TRAF3 and SGT1 in the serum found that the expressions of TRAF3 and SGT1 were significantly lower in septic patients than in HCs (**Figure 7**). These results suggest that decreased expression of TRAF3 and SGT1 as well as increased expression of IL-1β and IL-18 may be associated with the poor prognosis of patients with clinical sepsis, and this result is also consistent with our previous observation on the overall animal and cellular levels.

## Discussion

The NLRP3 inflammasome is a supramolecular complex that plays a key role in immune monitoring (24). Abnormal activation of inflammasomes has been linked to a number of human diseases, including gout, diabetes, and atherosclerosis (25–27). Nifuroxazide can reduce sepsis-related ALI and myocardial injury by inhibiting the NLRP3 inflammasome activation and reducing the inflammatory mediator secretion (28). Dysfunction of the NLRP3 inflammasome can lead to an increased inflammatory response in the body, thus aggravating the development of sepsis (29). Clarifying the regulatory mechanism of NLRP3 inflammasome activation through multiple signaling pathways and PTMs will help to identify more therapeutic molecules in sepsis.

HSFs are a family of evolutionary conserved DNA-binding proteins that regulate gene expression at the transcriptional level. The human genome encodes six heat shock transcription factor (HSF) proteins: HSF1, HSF2, HSF4, HSF5, HSFX, and HSFY. Currently, only HSF1 has been reported in sepsis-related studies. HSF1 activation is regulated by a multimolecular chaperone complex composed of HSP40, HSP70, and HSP90 (13, 30). It has been reported that inhibition of HSP70 expression may lead to dysfunction of immune cells and reduce resistance to infectious infection during severe sepsis. Under normal physiological conditions, HSF1 exists in the cytoplasm as an inactive monomer. Under stress, HSF1 is activated and transferred to the nucleus, where it binds to HSEs in the target protein promoter region to initiate transcription and ultimately protect cells from stress damage (31, 32). Our previous work has shown that HSF1 improved survival in mice with endotoxemia by inhibiting the inflammatory response (33). By inhibiting the expression of XCL-1/XCR-1 and MCP-1/CCR-2, two pairs of chemokine ligands/receptors, HSF1 inhibited the migration and infiltration of neutrophils and macrophages in lung tissue and finally alleviated LPS-induced ALI in mice (34). Studies have shown that HSF1 can reduce the activation of NLRP3 inflammasome in macrophages during alcohol-related liver injury, thereby reducing the alcohol-related liver injury (15). Whether HSF1 is involved in the activation of the NLRP3 inflammasome in septic ALI has not been reported yet. In the present study, we demonstrated for the first time that HSF1 inhibited the activation of the NLRP3 inflammasome and the secretion of IL-1β in septic ALI *via* suppressing the NF-κB signaling pathway and promoting the ubiquitination of NLRP3. This finding provides a novel mechanism for how HSF1 is involved in the intracellular immune response to protect against septic ALI.

Accumulating evidence suggests that HSF1 has developed many mechanisms to protect the immune system (35). Consistent with these studies, the present research provides a novel description for HSF1 protection of the immune system *via* targeting NLRP3 inflammasome. It was revealed conclusively that HSF1 inhibited the activation of the NLRP3 inflammasome and the secretion of IL-1β *via* suppressing the NF-κB signaling pathway and promoting the ubiquitination of NLRP3.

TRAF3 is an intracellular signaling molecule with important functions in signal transduction pathways (36). TRAF3 acts as an adaptor molecule. Other signaling proteins, such as CIAP and NIK, are recruited into protein complexes to control signal transduction pathways. These events include the activation of NF-κB and activator protein 1, key transcription factors that control many immune response genes (37, 38). Coronavirus ORF3a has been reported to activate the NLRP3 inflammasome by inducing pro-IL-1β transcription through activation of NF-κB, which is mediated by TRAF3-dependent ubiquitination (39). Therefore, the activation of the NLRP3 inflammasome can be reduced by regulating TRAF3 expression and then inhibiting the NF-κB signaling pathway. In sepsis, whether HSF1 can participate in the activation of the NLRP3 inflammasome by regulating the expression of TRAF3 is still unclear. In this study, we found that HSF1 upregulated TRAF3 expression to inhibit the NF-κB signaling pathway, thereby inhibiting the production of NLRP3 at the transcriptional level and ultimately inhibiting the activation of the NLRP3 inflammasome.

The ubiquitin system is a highly complex PTM system. It has been reported that the E3 ubiquitin ligase TRIM31 directly binds to NLRP3 to promote the proteasome degradation of NLRP3, thereby inhibiting the activation of the NLRP3 inflammasome, suggesting that ubiquitination is one of the main mechanisms regulating the activation of the NLRP3 inflammasome (40). SGT1, as a ubiquitin ligase-associated protein, can interact with a variety of proteins, including molecular chaperones and certain SCF ubiquitin ligases (41). Studies have shown that SGT1 can enhance the ubiquitination activity of SSPH2 *in vitro* to regulate innate immunity (42). Whether SGT1 is involved in HSF1-regulated ubiquitination of NLRP3 is still unclear. In this study, we found that there was an interaction between SGT1 and NLRP3, and HSF1 could promote the transcriptional expression of SGT1, thereby promoting the ubiquitination of NLRP3 and inhibiting the activation of NLRP3 inflammasome at the level of a PTM. However, its specific types of ubiquitination and ubiquitination sites are still unclear and need to be further studied.

**Figure 8:**
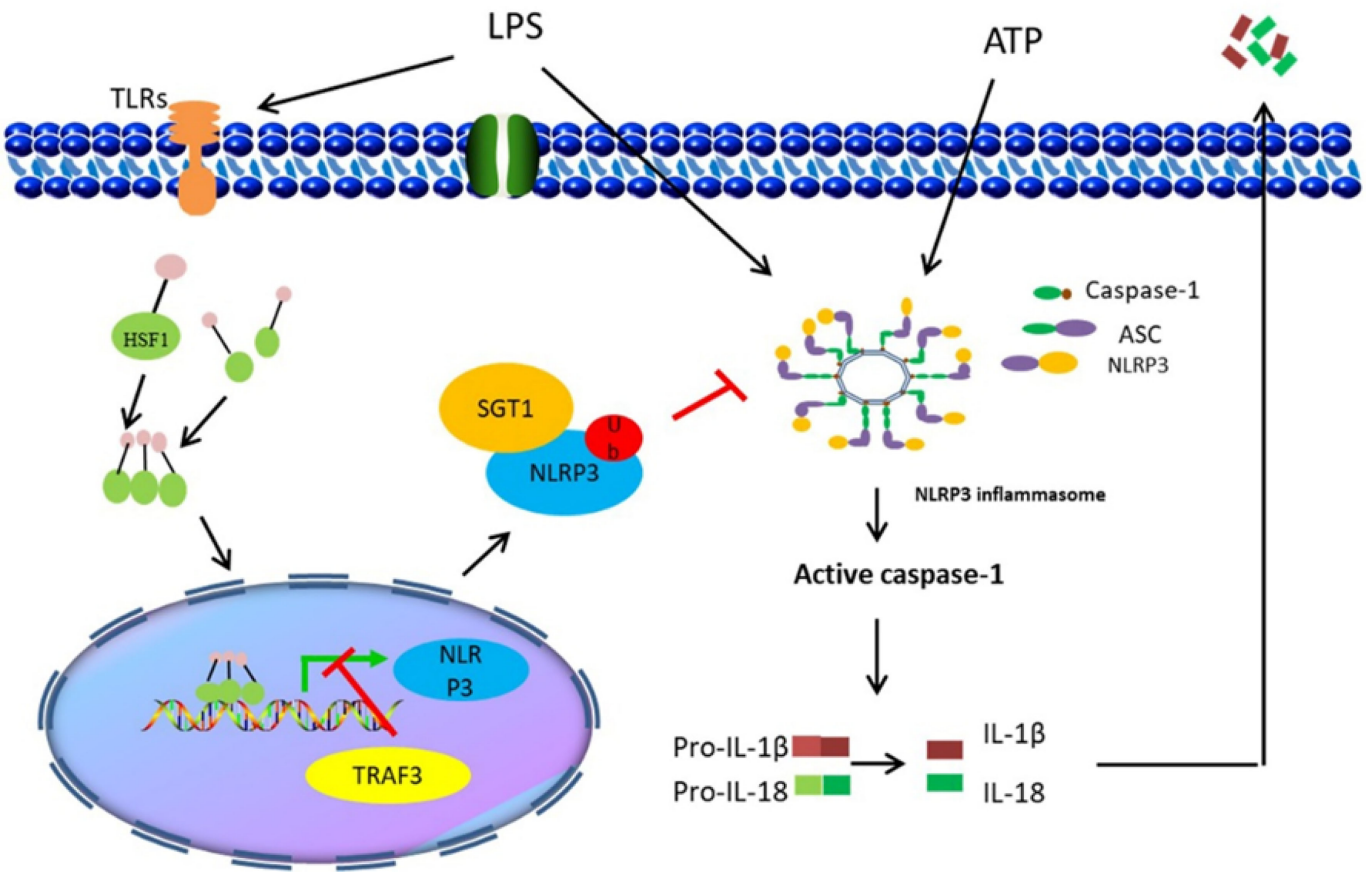
Illustration of HSF1 protecting sepsis-induced acute lung injury by inhibiting NLRP3 inflammasome activation.

Collectively, the findings of the present study demonstrated that HSF1 not only inhibited the development of ALI in sepsis but also suppressed the NLRP3 inflammasome activation and IL-1β maturation. The inhibitory activity was mediated by HSF1 and involved in the suppression of the NF-κB signaling pathway and promotion of NLRP3 ubiquitination (**Figure 8**). The findings of this study provided a more enhanced understanding of the interplay between HSF1 and immune response and revealed a novel mechanism of HSF1-mediated protection of septic ALI, which contributes to an out-of-control inflammatory response. Our data may also provide new therapeutic targets for the inflammatory response of septic ALI and related diseases.

### Clinical Perspectives

i) Uncontrolled inflammatory response and multiple organ dysfunction induced by sepsis are attributed to its high mortality rate. Therefore, inhibition of the inflammatory response can protect sepsis-induced ALI and improve the survival of sepsis.
ii) HSF1 inhibits NLRP3 inflammasome activation by upregulating TRAF3 and SGT1 expression at the transcriptional level and post-translational modification level, respectively, thereby alleviating septic ALI.
iii) The findings of this study revealed a novel mechanism of HSF1-mediated protection of septic ALI, which contributes to an out-of-control inflammatory response. Our data may also provide new therapeutic targets for the inflammatory response of septic ALI and related diseases.

## Data Availability Statement

The original contributions presented in the study are included in the article/supplementary material. Further inquiries can be directed to the corresponding authors.

## Ethics Statement

The studies involving human participants were reviewed and approved by the ethical review of the Third Xiangya Hospital of Central South University. The patients/participants provided their written informed consent to participate in this study. The animal study was reviewed and approved by the Institutional Animal Care and Use Committee of Central South University.

## Author Contributions

XS and TL assisted in the design of the study, performed the experiments, analyzed the data, and drafted the manuscript. YL, SCT, and LY contributed to the manuscript revision. LX and LF helped draft the manuscript. YZ, HC, KW, and XX helped perform some of the experiments and supporting materials. SPT and HZ contributed to the study design and revision of the manuscript and provided funding. All authors have read and approved the final manuscript.

## Funding

This study is supported by the National Natural Science Fund of China (Grant No. 81870071), National Natural Science Fund of China (Grant No. 82170095), Hunan Natural Science Fund of China (Grant No. 2019JJ40393), and Changsha Municipal Natural Science Foundation (Grant No. kq2014228).

## Conflict of Interest

The authors declare that the research was conducted in the absence of any commercial or financial relationships that could be construed as a potential conflict of interest.

## Publisher’s Note

All claims expressed in this article are solely those of the authors and do not necessarily represent those of their affiliated organizations, or those of the publisher, the editors and the reviewers. Any product that may be evaluated in this article, or claim that may be made by its manufacturer, is not guaranteed or endorsed by the publisher.

## Abbreviations

HSF1, heat shock factor 1; HSE, heat shock element; NLRP3, NOD-like receptor protein-3; CLP, cecal ligation and puncture; ALI, acute lung injury; ARDS, acute respiratory distress syndrome; MODS, multiple organ dysfunction syndrome; PRRs, pattern recognition receptors; TLRs, Toll-like receptors; NLRs, NOD-like receptors; LRR, leucine-rich repeat; PAMPs, pathogen-associated molecular patterns; DAMPs, danger-associated molecular patterns; LPS, lipopolysaccharide; TPR, tetratricopeptide repeat; EMSA, electrophoretic mobility shift assay; Co-IP, co-immunoprecipitation; BALF, bronchoalveolar lavage fluid; IL-18, interleukin-18; IL-1β, interleukin-1β.
